# Associations of Mediterranean diet with psychological ill-being and well-being throughout the pregnancy course: The GESTAFIT project

**DOI:** 10.1007/s11136-022-03121-2

**Published:** 2022-03-16

**Authors:** Marta Flor-Alemany, Laura Baena-García, Jairo H. Migueles, Pontus Henriksson, Marie Löf, Virginia A. Aparicio

**Affiliations:** 1 Department of Physiology, School of Pharmacy, University of Granada, Granada, Spain; 2 Institute of Nutrition and Food Technology, University of Granada, Granada, Spain; 3 Sport and Health University Research Institute (iMUDS), Granada, Spain; 4 Department of Nursing, Faculty of Health Sciences, University of Granada, Ceuta, Spain; 5 Department of Health, Medicine and Caring Sciences, Linköping University, Linköping, Sweden; 6 PROFITH “Promoting FITness and Health Through Physical Activity” Research Group, Sport and Health University Research Institute (iMUDS), Department of Physical Education and Sports, Faculty of Sport Sciences, University of Granada, Granada, Spain; 7 Department of Biosciences and Nutrition, Karolinska Institutet, Huddinge, Sweden

**Keywords:** Anxiety, Depression, Diet, Mediterranean, Mental Health, Pregnancy, Pregnant women

## Abstract

**Purpose:**

The relation between diet and maternal mental health during pregnancy might be relevant to prevent adverse materno-foetal outcomes. This study examined the association of Mediterranean diet (MD) adherence and MD components with mental health during pregnancy.

**Methods:**

This secondary analysis of the GESTAFIT trial included longitudinal data from 152 pregnant women. Dietary habits were assessed with a food frequency questionnaire, and MD adherence was derived from it using the Mediterranean Food pattern. Psychological ill-being (i.e., negative affect, anxiety, and depression) and well-being (i.e., emotional intelligence, resilience, positive affect) were assessed with the Spanish version of well-established self-reported questionnaires. Cross-sectional (16th gestational week [g.w.]) and longitudinal associations (34th g.w.) between MD and mental health were studied using linear regression models.

**Results:**

A greater MD adherence was inversely associated with negative affect and anxiety; and positively associated with emotional regulation, resilience and positive affect at the 16th and 34th g.w. (|β| ranging from 0.179 to 0.325, all *p* < 0.05). Additionally, a higher intake of whole grain cereals, fruits, vegetables, fish, olive oil and nuts, and a lower intake of red meat and subproducts and sweets were associated with lower negative affect, anxiety, depression and higher emotional regulation, resilience and positive affect throughout gestation (|β| ranging from 0.168 to 0.415, all *p* < 0.05).

**Conclusion:**

A higher intake of whole grain cereals, fruits, vegetables, fish, olive oil and nuts, together with a lower intake of red meat and sweets, resulted in a higher MD adherence, which was associated with a better mental health during pregnancy.

**Supplementary Information:**

The online version contains supplementary material available at 10.1007/s11136-022-03121-2.

## Introduction

Pregnancy is a major life event that entails biological, psychological, and social changes in the women’s mental health [1]. Depression and anxiety are the most prevalent mental health disorders during pregnancy [2]. Current data indicates that 26–31% of pregnant women are at risk of depression in the second trimester [3], 7–15% suffer from antenatal depression [4], and 14–54% from antenatal anxiety [5]. These mental health disorders seem to increase the risk for pregnancy-related complications (e.g., preeclampsia, spontaneous preterm delivery or low birth weight) [2, 4, 5].

Therefore, identifying protective factors for mental health in pregnant women is warranted [6]. Both, low levels of psychological ill-being and high levels of well-being should be considered to reach an optimal metal health status [7]. The dietary intake during pregnancy might affect the psychological ill-being and well-being in pregnant women [8, 9]. Previous research found that the intake of certain food groups and nutrients (i.e., refined grains, sweets, energy drinks, and fast foods) increases the risk for antenatal depressive symptoms compared with alternative healthy choices (i.e., fruits, vegetables, fish and whole grains) [9–11]. Notwithstanding, there is a shift in the nutrition field towards assessing the whole diet and its quality to investigate the diet-disease relationship [8]. As an example, the Mediterranean Diet (MD, characterized by a high intake of fruits, vegetables, whole grains, fiber, olive oil, and low intake of red meat, dairy, and processed foods) is associated with a lower risk of depression in the general population [12], yet information in pregnant women is scarce.

Thus, research investigating not only single food groups, but also the diet quality during pregnancy (e.g., MD), is required to provide robust evidence on the association of diet with psychological ill-being and well-being. The aim of this study was to analyze the association of dietary habits and MD adherence at the 16th gestational week (g.w.) with psychological ill-being and well-being at the 16th and the 34th g.w.

## Methods

### Study design and participants

This longitudinal study was conducted in Granada (Spain), within the GEStation and FITness (GESTAFIT) project framework, where a concurrent training program from the 17th week until delivery (3 days/week, 60 min/session) was conducted [13]. It consisted in a combination of aerobic-resistance exercises of moderate-to-vigorous intensity. Each exercise session included a 10-min warm-up period with walks, mobility and activation exercises. The main part of the first and last weekly sessions consisted of 40 min of exercises organized in two resistance circuits of 15 exercises (40″ work/20″ rest), alternating with cardiovascular blocks (concurrent training). The second session of the week was focused on aerobic training through dancing, proprioceptive and coordinative circuits, and interval walks. The study was carried out at the “Sport and Health University Research Institute” (Granada, Spain), and at the “San Cecilio and Virgen de las Nieves University Hospitals” from November 2015 to April 2018. A total of 159 women met the inclusion–exclusion criteria (Supplementary Table S1). Fourteen of these women were either younger than 25 (*n* = 6) or older than 40 years old (*n* = 8). Among them, 152 participants were included upon providing data in sociodemographic characteristics and MD adherence at the 16th g.w. (Supplementary Figure S1). Women provided a written informed consent. The study was approved by the Clinical Research Ethics Committee of Granada, Government of Andalusia, Spain (code: GESFIT-0448-N-15).

### Sociodemographic characteristics

Sociodemographic characteristics (i.e., age, number of miscarriages and educational and working status) were compiled with a self-reported questionnaire (anamnesis) at the 16^th^ g.w.

### Sample size calculation

The sample size for this study depended on the ‘a priori’ analyses of the statistical power performed in the GESTAFIT project [13]. Based on the primary outcome (i.e., maternal weight gains and maternal/neonatal glycemic profile), we planned to recruit 60 women assuming a statistical power of 90%, α = 0.05, and a 15% of potential withdrawals. Given the exploratory basis of the present study (secondary outcomes) we did not calculate the sample size. Notwithstanding, we performed an “a posteriori” analysis to investigate the effect sizes detectable in this study. Assuming a statistical power of 80% and 5% type I error, we have enough sample to detect small-to-medium standardized association sizes at the 16th g.w. (*n* ≥ 117, minimum detectable f^2^ = 0.12) and at the 34th g.w. (*n* ≥ 109, minimum detectable *f*^2^ = 0.13) [14].

### Psychological well-being

Positive affect (the extent to which a person feels enthusiastic, active, and alert) was assessed with the State Positive Affect Schedule (PANAS-S) [15, 16]. This questionnaire is comprised of 10 positive and 10 negative emotional states that are answered on a 5-point Likert scale (1–5). The total score ranges from 10 to 50 with higher scores reflecting greater affective well-being (PANAS-S positive subscale). The PANAS is one of the most widely used measures of affectivity and has been reported to have excellent psychometric properties in the adult population [15–17].

The 3 subscales of The Trait Meta-Mood Scale (TMMS) [18] were employed to assess emotional attention (i.e., to what extent individuals tend to observe and think about their feelings and moods), emotional clarity (i.e., the understanding of one's emotional states) and emotional regulation (i.e., individuals’ beliefs about ability to regulate their feelings). The Spanish modified version of the TMMS [18] had appropriate reliability and validity. Each subscale is comprised of 8 items which are rated on a 5-point Likert scale (1–5). Therefore, the total scores range from 8 to 40, with higher scores reflecting greater attention, clarity, and regulation.

The Connor-Davidson Resilience Scale (CD-RISC) was employed to assess resilience to stress (i.e., individual’s ability to thrive despite adversity) [19]. The CD-RISC consists of 10 items which are score from 0 to 4. Therefore, the total score ranges from 0 to 40, with higher scores indicating a greater resilience. In regard to reliability, the CD-RISC showed good psychometric properties in young adults; with a Cronbach’s α of 0.85 [19].

### Psychological ill-being

Negative affect (a variety of aversive mood states, including anger, contempt, disgust, guilt, fear, and nervousness) was assessed with the PANAS-S negative subscale [15, 16]. The total score ranges from 10 to 50 with higher scores reflecting greater emotional distress.

The State Trait Anxiety Index (STAI-S) questionnaire was employed to evaluate state-anxiety levels in pregnant women at the moment of the evaluation [20, 21]. It consists of 20 items on a four-point scale (0–3). The total score ranges from 0 to 60 with higher values indicating greater levels of anxiety.

The validated 20-item Spanish version of the Center for Epidemiological Studies Depression Scale (CES-D), the most accepted calculation method across the literature, was employed to assess pregnant antenatal depression [22]. The CES-D is composed of 20 items; each one scored in a scale from ‘‘0’’ to ‘‘3’’, thus the total score varies from 0 to 60. In regard to reliability, it has very good internal consistency (Cronbach’s α = 0.91) with similar coefficients by groups of age and sex and by interviewer [22]. In this study, we applied the cut-off point of 16 to dichotomize the group into having or not risk of clinical depression as previously done in pregnant women [6].

### Low back pain

Low back pain was assessed with the Pain Visual Analogue Scale[23]. The score is determined by measuring the distance on the 100-mm line between the “no pain” anchor and the participant’s mark.

### Dietary assessment and Mediterranean diet adherence

A food frequency questionnaire validated in Spanish non-pregnant adult population [24] was used to assess dietary habits at the 16th and 34th g.w. in face-to-face interviews by trained staff. The Mediterranean Food Pattern [25] was derived from the food frequency questionnaire following previously-defined standards. The Mediterranean Food pattern consists in a quintile-based sum score of eight food groups (olive oil, fiber, fruits, vegetables, fish, cereals, meat, and alcohol) and it ranges from 5 to 40 (higher scores indicate higher MD adherence). Alcohol consumption was not considered since pregnant women must not drink alcohol. Thus, our score consisted of seven elements and it ranged from 4 to 35. The present study only targeted women in the second trimester of pregnancy (13th to 27th g.w.), where dietary habits are relatively more constant, being more representative of dietary behaviour across the whole gestational period [26]. Moreover, we previously observed that the MD adherence and dietary habits remained unchanged in our participants between the 16th g.w. and the 34th g.w. [27]. Consequently, the dietary pattern at the 16th g.w. was considered representative of the pregnancy period.

### Statistical analysis

Descriptive statistics were summarized as mean (standard deviation) or frequency (%) as appropriate. Linear regression analyses were employed to study the associations of MD adherence (at the 16th g.w.) with psychological ill-being and well-being (at the 16th and the 34th g.w.). Covariates were selected based on exploratory analyses performed in a previously published study involving this study sample [28]. Two models were conducted, Model I was unadjusted and Model II was adjusted for age, educational status, number of miscarriages and low back pain. The number of miscarriages have been previously associated with anxiety and depressive symptoms during pregnancy [29]. Thus, we investigated the influence of this covariate in our models by (1) comparing the psychological ill-being and well-being outcomes between women with at least one miscarriage and women without any by a one-way analysis of covariance (ANCOVA) after adjusting for age, educational status and low back pain (at the 16th g.w.) and the exercise intervention (at the 34th g.w.); and (2) exploring the interaction between number of miscarriages (0 = no miscarriages and 1 = one or more miscarriages) and the MD adherence on psychological ill-being and well-being during pregnancy. Since the number of miscarriages*MD adherence interaction term was not significant (all *p*’s > 0.2) we decided not to conduct separate models for women with no miscarriages or one or more miscarriages. The Model II in the longitudinal analysis (i.e., MD at 16th g.w. and mental health at 34th g.w.) was additionally adjusted for the group allocation to account for the exercise intervention delivered in the GESTAFIT project. Moreover, since the associations between MD adherence, ill-being and well-being might be affected by baselines values the Model III in the longitudinal analyses was additionally adjusted by baseline values (i.e., ill-being and well-being indicators at the 16th g.w.). Furthermore, we investigated the group allocation*MD adherence interaction term, which was not significant (all *p*’s > 0.2), and thus we decided not to conduct separate models for women in the control and intervention groups. Additional sensitivity analyses were conducted only in the control group (n: 46–53 depending on the outcome) and also excluding those women who did not meet the age inclusion criteria (*n* = 14) and results remained the same.

Finally, linear regression models were employed to explore the associations between single food groups and those mental health indicators associated with the MD adherence. Cross-sectional and longitudinal associations with the abovementioned covariates were performed.

The Benjamini–Hochberg procedure [23] was applied to account for the random effect in multiple comparisons for all the tests included in the primary analysis (i.e., MD adherence associations with mental health indicators at the 16th and 34th g.w.) and separately for all the tests included in the MD components analysis (i.e., MD components associations with mental health indicators at the 16th and 34th g.w.) with *q* = 0.05 (false discovery rate).

All analyses were conducted using the Statistical Package for Social Sciences (IBM SPSS Statistics for Windows, version 22.0, Armonk, NY) and the level of significance was set at *p* ≤ 0.05.

## Results

Among the 159 pregnant women participating in the GESTAFIT project, 152 provided valid data on MD adherence and sociodemographic characteristics (Supplementary Figure S1). Psychological ill-being and well-being, and clinical and sociodemographic characteristics of study participants are shown in Table 1. Briefly, most participants (59%) presented a high educational status (i.e., university), were married or with partner (59%), were working (68%), and did not have any miscarriage in the past (59%). Around 26% of women were at risk of clinical depression at the 16th g.w. and 38% at the 34th g.w.

**Table 1:** Psychological ill-being and well-being, and clinical and sociodemographic characteristics of study participants (*n* = 152)

Variable	*n*	Mean (SD) or *n* (%)
16th gestational week		
Age (years)	152	32.9 (4.6)
Low back pain (VAS)	152	22.2 (24.5)
*Educational Status, n (%)*	152	
Low educational status		17 (11.2)
Medium educational status		45 (29.6)
High educational status		90 (59.2)
*Marital status, n (%)*	152	
Married/with partner		90 (59.2)
Divorced/Single/widow		62 (40.8)
*Working status, n (%)*	152	
Working		104 (68.4)
Not working		48 (31.6)
*Number of miscarriages, n (%)*	152	
0		89 (58.6)
1		44 (28.9)
2		16 (10.5)
3 or more		3 (2.0)
Mediterranean diet adherence (4–35)	152	20.6 (5.0)
*Psychological ill-being*		
Negative Affect (PANAS-S, 10–50)^a^	141	17.3 (6.7)
Anxiety (STAI-S, 20–80)^b^	140	14.2 (9.0)
Depression risk score (CES-D, 0–60)^c^	117	11.2 (8.1)
Depression (yes)^d^ (n%)	117	30 (25.6)
*Psychological well-being*		
Emotional Attention (TMMS-A, 8–40)^e^	142	25.39 (6.2)
Emotional Clarity (TMMS-C, 8–40)^f^	142	30.56 (4.9)
Emotional Regulation (TMMS-R, 8–40)^g^	142	30.02 (5.2)
Resilience (CD-RISC, 0–40)^h^	138	30.21 (5.2)
Positive Affect (PANAS-S, 10–50)^i^	141	34.33 (6.6)
34th gestational week		
*Psychological ill-being*		
Negative Affect (PANAS-S, 10–50)^a^	115	18.62(7.0)
Anxiety (STAI-S, 20–80)^b^	109	17.0 (10.9)
Depression risk score (CES-D, 0–60)^c^	117	13.27 (7.7)
Depression (yes)^d^ (n%)	117	44 (37.6)
*Psychological well-being*		
Emotional Attention (TMMS-A, 8–40)^e^	119	25.60 (5.9)
Emotional Clarity (TMMS-C, 8–40)^f^	119	30.38 (5.3)
Emotional Regulation (TMMS-R, 8–40)^g^	119	30.11 (5.1)
Resilience (CD-RISC, 0–40)^h^	112	30.08 (5.1)
Positive Affect (PANAS-S, 10–50)^i^	115	33.0 (7.6)

Values shown as mean (standard deviation) unless otherwise is indicated

*SD* standard deviation, *VAS* visual analogue scale

^a^Higher scores reflect greater emotional distress

^b^Higher values reflect greater levels of anxiety

^c^Higher scores indicate the presence of more depressive symptomatology

^d^Cut-off score of 16 is indicative of potential depression

^e^Higher scores reflect greater attention

^f^Higher scores reflect greater clarity

^g^Higher scores reflect greater regulation

^h^Higher scores indicate a greater resilience

^i^Higher scores reflect greater affective emotional health/experience

The cross-sectional associations between MD adherence and psychological ill-being and well-being indicators (at the 16th g.w.) are shown in Table 2. In model II (adjusted model), MD adherence was inversely associated with anxiety (β =  − 0.200, SE = 0.086, *p* = 0.022) and we observed a borderline non-significant association with depression (β = -0.181, SE = 0.098, *p* = 0.066). Regarding well-being, MD adherence was positively associated with emotional regulation (β = 0.179, SE = 0.088, *p* = 0.043), resilience (β = 0.206, SE = 0.089, *p* = 0.022) and positive affect (β = 0.182, SE = 0.082, *p* = 0.029).

**Table 2:** Cross-sectional and longitudinal associations of Mediterranean diet adherence with psychological ill-being and psychological well-being

Mental health indicators	n	Cross-sectional (16th g.w.)				n	Longitudinal (16th vs 34th g.w.)				n		
		Model I		Model II			Model I		Model II^a^			Model III	
		β	p	β	p		β	β	β	p		β	p
*Psychological ill-being*													
Negative affect	141	− 0.149	0.077	− 0.130	0.150	115	− 0.224	**0.016**	− 0.241	**0.014**	111	− 0.183	**0.026**
Anxiety	140	− 0.205	**0.015**	− 0.200	**0.022**	109	− 0.295	**0.002**	− 0.325	**0.001**	105	− 0.172	**0.040**
Depression	117	− 0.229	**0.013**	− 0.181	0.066	117	− 0.184	**0.048**	− 0.171	0.066	91	0.078	0.403
*Psychological well-being*													
Emotional attention	142	− 0.023	0.790	− 0.016	0.860	119	− 0.162	0.078	− 0.106	0.263	114	− 0.055	0.450
Emotional clarity	142	0.129	0.125	0.114	0.203	119	0.167	0.073	0.121	0.212	114	0.087	0.319
Emotional Regulation	142	0.202	**0.016**	0.179	**0.043**	119	0.306	**0.001**	0.295	**0.001**	114	0.171	**0.041**
Resilience	138	0.191	**0.025**	0.206	**0.022**	112	0.275	**0.003**	0.259	**0.012**	107	0.120	0.145
Positive affect	141	0.144	0.089	0.182	**0.029**	115	0.202	**0.030**	0.185	**0.048**	111	0.070	0.369

Model I was unadjusted. Model II was adjusted for age, educational status, number of miscarriages, low back pain

^a^Model II in the longitudinal analysis was additionally adjusted for exercise intervention

Model III in the longitudinal analysis was additionally adjusted for baseline values (i.e., mental health indicator at the 16th gestational week)

Boldface indicates those outcomes which surpassed the multiple comparison test

Longitudinal associations of MD adherence with mental health indicators (at the 34th g.w.) are presented in Table 2. The adjusted model (Model II) showed that MD adherence was inversely associated with negative affect (β =  − 0.241, SE = 0.096, *p* = 0.014) and anxiety (β =  − 0.325, SE = 0.098, *p* = 0.001), and we observed a borderline non-significant association with depression (β =  − 0.171, SE = 0.092, *p* = 0.066). Furthermore, MD adherence was positively associated with emotional regulation (β = 0.295, SE = 0.089 *p* = 0.001), resilience (β = 0.259, SE = 0.101, *p* = 0.012), and positive affect (β = 0.185, SE = 0.092, *p* = 0.048). The associations between MD, negative affect (β =  − 0.183; SE = 0.081, *p* = 0.026), anxiety (β =  − 0.172; SE = 0.083, *p* = 0.040) and emotional regulation (β = 0.171; SE = 0.083, *p* = 0.041) remained significant after adjusting by baseline values (Model III). After correcting for multiplicity, we observed that the cross-sectional and longitudinal associations between MD adherence and mental health indicators remained significant.

The cross-sectional associations of single food groups with psychological ill-being and well-being (at the 16th g.w.) after adjusting for the above-mentioned covariates are shown in Table 3. A higher intake of whole-grain cereals, fruits, vegetables, fish and nuts, and a lower intake of red meat and subproducts and sweets was associated with lower negative affect, anxiety and depression and greater emotional regulation, resilience and positive affect (|β| ranging from 0.168 to 0.268, all *p* < 0.05). After correcting for multiplicity, we observed that the associations between vegetables, resilience and positive affect and the associations between fish, nuts and positive affect remained significant. The longitudinal associations of single food groups with psychological ill-being and well-being (at the 34th g.w.) after adjusting for the above-mentioned covariates are shown in Table 4. A higher intake of fruits, olive oil and nuts together with a lower intake of red meat and subproducts was associated with lower negative affect, anxiety and depression and greater emotional regulation, resilience and positive affect (|β| ranging from 0.205 to 0.415, all *p* < 0.05). After correcting for multiplicity, we observed that the associations fruits, negative affect, anxiety, depression and emotional regulation remained significant. Additionally, the associations between red meat, anxiety and resilience and the associations between olive oil, nuts and resilience remained significant.

**Table 3:** Cross-sectional association of single food groups, psychological ill-being and psychological well-being at the 16th gestational week

N	Psychological ill-being						Psychological well-being					
	Negative Affect		Anxiety		Depression		Emotional Regulation		Resilience		Positive Affect	
	141		140		117		142		138		141	
Single food groups	β	p	β	p	β	p	β	p	β	p	β	p
Whole grain cereals (s/wk)	−0.183	0.035	−0.164	0.052	−0.172	0.065	0.041	0.634	−0.041	0.645	−0.038	0.640
Potatoes (s/wk)	0.081	0.366	0.055	0.527	0.021	0.823	−0.036	0.675	−0.042	0.637	−0.065	0.427
Fruits (s/wk)	−0.194	0.039	−0.182	0.036	−0.228	0.022	0.185	0.035	0.060	0.507	0.130	0.112
Vegetables (s/wk)	−0.008	0.928	−0.097	0.252	−0.059	0.540	0.168	0.048	0.268	**0.002**	0.244	**0.002**
Pulses (s/wk)	0.082	0.350	−0.019	0.828	0.026	0.786	0.109	0.202	0.044	0.622	0.109	0.179
Fish (s/wk)	0.025	0.777	0.064	0.459	0.032	0.738	0.053	0.543	0.052	0.561	0.213	**0.008**
Red Meat and subproducts (s/wk)	0.141	0.103	0.140	0.098	0.238	0.009	0.002	0.983	−0.084	0.337	0.004	0.963
Poultry (s/m)	0.032	0.718	−0.007	0.930	−0.113	0.226	−0.001	0.988	−0.055	0.535	0.097	0.227
Dairy products(s/wk)	0.033	0.708	−0.038	0.655	0.006	0.946	−0.010	0.909	0.036	0.683	−0.038	0.634
Olive Oil (s/wk)	0.005	0.950	−0.030	0.724	−0.090	0.328	−0.017	0.837	0.061	0.481	−0.033	0.678
Nuts (s/wk)	0.032	0.715	−0.007	0.931	0.037	0.693	0.190	0.025	0.170	0.055	0.220	**0.006**
Sweets (s/wk)	0.183	0.045	0.220	0.012	0.140	0.150	−0.032	0.722	−0.066	0.471	0.035	0.679

Analyses were adjusted for age, educational status, number of miscarriages and low back pain. S, servings; wk, week

Boldface indicates those outcomes which surpassed the multiple comparison test

**Table 4:** Longitudinal association of single food groups, psychological ill-being and psychological well-being at the 34th gestational week

N	Psychological ill-being						Psychological well-being					
	Negative affect		Anxiety		Depression		Emotional regulation		Resilience		Positive affect	
	115		109		117		119		112		115	
Single food groups	β	p	β	p	β	p	β	p	β	p	β	p
Whole grain cereals (s/wk)	− 0.165	0.093	− 0.129	0.229	− 0.099	0.288	0.070	0.470	− 0.051	0.611	− 0.064	0.497
Potatoes (s/wk)	− 0.023	0.815	0.083	0.410	0.170	0.067	− 0.082	0.402	0.018	0.865	− 0.007	0.942
Fruits (s/wk)	− 0.357	** < 0.001**	− 0.415	** < 0.001**	− 0.365	** < 0.001**	0.278	**0.004**	0.205	0.045	0.243	0.010
Vegetables (s/wk)	0.059	0.538	− 0.003	0.977	− 0.040	0.663	0.027	0.778	0.140	0.162	0.135	0.139
Pulses (s/wk)	0.084	0.390	0.005	0.957	0.111	0.234	0.027	0.780	0.025	0.801	0.126	0.174
Fish (s/wk)	− 0.053	0.588	− 0.051	0.622	0.025	0.787	0.107	0.289	0.034	0.735	0.102	0.276
Red Meat and subproducts (s/wk)	0.086	0.373	0.237	**0.016**	0.143	0.116	0.021	0.828	− 0.234	**0.015**	− 0.163	0.075
Poultry (s/m)	0.099	0.309	0.043	0.670	− 0.050	0.591	− 0.007	0.936	− 0.104	0.292	0.089	0.336
Dairy products(s/wk)	0.074	0.450	0.049	0.631	0.031	0.737	− 0.056	0.548	− 0.016	0.875	0.106	0.260
Olive Oil (s/wk)	− 0.108	0.253	− 0.142	0.153	− 0.006	0.944	0.108	0.229	0.304	**0.001**	0.014	0.874
Nuts (s/wk)	− 0.206	0.043	− 0.231	0.026	0.040	0.681	0.210	0.027	0.247	**0.017**	0.173	0.076
Sweets (s/wk)	0.101	0.326	0.009	0.932	0.151	0.112	− 0.019	0.846	− 0.032	0.758	− 0.016	0.867

Analyses were adjusted for age, educational status, number of miscarriages, low back pain and exercise intervention. S, servings; wk, week

Boldface indicates those outcomes which surpassed the multiple comparison test

Additional sensitivity analyses (i.e., longitudinal associations of MD adherence at the 16th g.w. with mental health indicators at the 34th g.w.) showed similar results when exclusively including the control group participants in the analyses (Supplementary Table S2). Differences in psychological ill-being and psychological well-being of pregnant women at the 16th and 34th g.w. by number of miscarriages are shown in Supplementary Table S3. No differences were found in psychological ill-being and psychological well-being in women with no miscarriages or one or more miscarriages (all, *p*’s > 0.05).

## Discussion

Our results suggest that MD adherence during gestation is associated with lower negative affect, anxiety, and depression; and with greater emotional regulation, resilience, and positive affect during pregnancy. These associations seem to be driven by a higher intake of whole grain cereals, fruits, vegetables, fish, olive oil and nuts, and a lower intake of red and processed meat and sweets.

Women are at increased risk of experiencing mental health problems during pregnancy that can impact theirs’ and the infant’s health [4, 5]. The number of previous miscarriages have been shown to exert a negative influence on anxiety and depressive symptoms during pregnancy [29]. Recent evidence showed that the risk of miscarriages ranges from 12% in women aged 20–29 years increasing to 65% in women aged 45 years and older. The average population prevalence of women who have had one or more previous miscarriages was 41% which is within the range of estimated miscarriage risk given that pregnant women in the present study aged 21–44 years old [30]. This percentage of miscarriages might be considered high when only comparing with women within the same mean age (i.e., 30–34 years; miscarriage risk = 14%) [30]. In this sense, our analyses were adjusted for this covariate to account for the possible effect on mental health indicators, yet we did not observe any associations between number of miscarriages and mental health throughout the pregnancy course. Additionally, we did not observe any differences in psychological ill-being and well-being based on the number of miscarriages, neither did the miscarriages moderated the association of MD adherence with psychological health. Thus, we strongly believe that the number of miscarriages is not affecting the results reported in this study. We observed that 26% of our sample were at risk of depression at the 16th g.w., and this proportion increased to 38% at the 34th g.w., which agrees with previous estimations [3, 31]. MD adherence may exert a beneficial effect on mental health outcomes in adults, such as depressive symptoms, cognitive status and quality of life, altogether improving the brain health [12]. However, research regarding MD adherence and mental health during pregnancy is limited. Previous studies in pregnancy mainly focus on the associations of diet quality with depressive symptoms [8, 32–35], and with other psychological ill-being indicators to a lesser extent (e.g., stress and negative affect [11, 36, 37]). For instance, maternal dietary patterns similar to the MD (i.e., rich in vegetables, fruits, pulses, fish and nuts, among other components) were associated with lower depression during pregnancy [32, 33]. Likewise, Paskulin et al. [34] found that pregnant women with a low intake of fruits, beans and with high “common-Brazilian” dietary pattern composed of foods popular in Brazilian culture, such as rice or noodles, French rolls, beans, boneless beef/chicken or eggs, coffee with sugar, margarine, and artificial juices had higher prevalence of mental disorders (including depression and anxiety). Fowles et al. [11] found that women with diet quality below the median (i.e., Diet quality index) had higher depressive symptoms and stress than women above the median. Additionally, levels of depression tend to increase throughout pregnancy [38], and a recent study [32] suggested that the diet-mental health association might exist along the pregnancy course.

By virtue of the repeated measurements, our findings add evidence to the literature showing that MD adherence was associated with lower anxiety at the 16th and 34th g.w., and with less negative affect at the 34th g.w. Therefore, according to our results, adherence to a MD may attenuate the experience of negative affect especially in the third trimester of pregnancy when women generally suffer more stress and anxiety. Lindsay et al. [37] found no associations between MD adherence and negative affect during early-mid pregnancy (12th-20th g.w.). Given that psychological ill-being fluctuates during pregnancy [2], the lack of association between MD and negative affect found by Lindsay et al. [37] is not generalizable to the third trimester of pregnancy when we did find such association. A systematic review [36] showed an inverse association between dietary patterns comprised of whole foods, fruits, vegetables, fish and seafood (which happens to be characteristic of MD) and perinatal anxiety and depression, which agrees with our findings. Moreover, we found that a greater intake of whole grain cereals and fruits, and a lower intake of red meat and subproducts and sweets was associated with lower negative affect, anxiety, and depression during gestation. These findings are in agreement with previous studies in pregnant [35] and non-pregnant women [39], and in the general population [40, 41]; and could be explained by the fact that these predominant nutrients in these food groups (i.e., saturated fats and sugar) have pro-inflammatory effects when consumed in excess [42].

Pregnancy is a period during which psychological well-being often declines [43]. Current evidence supports the importance of the MD for the well-being in the non-pregnant adult population [44]. Micronutrient deficiencies including iron, zinc, folate, vitamin D and, particularly, essential fatty acids seem to affect the well-being in pregnancy [45], yet the evidence on the MD adherence and well-being during pregnancy is scarce [43]. In this regard, we found that MD adherence was related to well-being indicators at the 16th and 34th g.w., suggesting that MD may improve well-being throughout the pregnancy course. This relation could be explained by the synergistic combination of single nutrients that are positively linked to mental health. These nutrients include those which are protective against oxidative stress such as the monounsaturated fatty acids present in the olive oil, the polyunsaturated fatty acids in fish, the folate and B vitamins in fruits, vegetables, nuts and legumes [46]. Ferrer-Casales et al. [47] found that omega-3 fatty acids, present in fish, nuts, and grains, and the B vitamins found in fruits and vegetables, are the most important nutrients for the central nervous system functioning (e.g., neurotransmission, gene expression, and mood). This may explain our results on the association of higher intakes of fruits, vegetables, nuts and olive oil with lower psychological ill-being and higher resilience, emotional regulation, and positive affect. Of note, after adjusting for baseline values (i.e., mental health indicator at the 16th g.w.) the associations between MD, resilience and positive affect became non-significant. This means that the potential effect of MD on resilience and positive affect is not observable when considering the baseline levels of these indicators. Future studies with larger sample sizes should further explore this association to elucidate whether MD might be associated in pregnant women with certain levels on these variables.

The potential biochemical and physiological mechanisms underlying the association between diet and mental health are poorly understood. The literature has suggested the role of dietary factors in the monoamine synthesis, inflammation processes, hypothalamic–pituitary–adrenal axis regulation, and neurogenesis [48]. Additionally, diet can promote the production and secretion of brain-derived neurotrophic factor (BDNF), a peptide implicated in synaptic plasticity and neuronal survival, whose levels are decreased in pregnant women with depression [49]. Previous evidence shows that MD adherence is associated with lower levels of pro-inflammatory cytokines that inhibit the BDNF expression [50].

Furthermore, recent evidence has focused on the influence of gut microbiota on emotional behaviour, neurological processes and symptoms of both depression and anxiety [51, 52]. The gut microbiota is strongly affected by diet [52]; thus, specific dietary patterns (or even single food groups) might prevent mental disorders by changes in the microbiota composition and function [51]. A “healthy” dietary pattern (such as the MD) contains a larger amount of fruits, vegetables, and wholegrains, a rich source of prebiotics such as fermentable carbohydrates, polyols, and phytochemicals which promoted the growth and activity of beneficial bacteria [53]. MD during pregnancy has been associated with increased maternal gastrointestinal tract microbial diversity [54]. Increased consumption of fruits, vegetables and legumes with low red meat consumption were the key components driving this association [54]. In this line, we found that a greater MD adherence was associated with lower negative affect, anxiety, and depression during gestation. Similarly, the dietary factors associated with lower negative affect, anxiety, and depression in our study sample (i.e., wholegrain cereals, fruits and nuts), are protective of the microbiota and the mucous layer, leading to an anti-inflammatory environment [55]. Contrarily, red meat and sugar were associated with higher anxiety and depression, which seems plausible since they are likely to interrupt the normal function of the gut-brain, induce mucus loss and microbiota disturbance, leading to a pro-inflammatory environment [55].

## Limitations and strengths

The findings and implications of this study should be interpreted with a number of limitations in mind. First, the observational design does not allow a clear cause–effect identification. Second, the results should be interpreted cautiously, as we could be limited to detect small association sizes. Larger studies should further explore these associations in order to corroborate our results. Third, the participating women were enrolled in an exercise intervention that might affect our findings. However, we investigated the potential interaction between exercise and diet, we included the group allocation as confounder in our longitudinal analyses, and we further performed sensitivity analyses exclusively in the control group, all analyses suggesting no effect of the intervention on our findings. Fourth, the missing data in our study could bias our findings. Similar dropout rates have occurred in previous studies in pregnant women [56], and we found no differences in the baseline characteristics of the drop-outs and the completers. Fifth, although the questionnaires used to assess mental health were valid and reliable for the general population, their psychometric properties have not been extensively tested during pregnancy, except for the STAI which is validated in pregnant women [20]. However, all the questionnaires employed in the present study to assess psychological ill-being and well-being have been previously employed in pregnant women [6, 28]. Future studies should investigate the validity of the mental health questionnaire in pregnant women. Additionally, although our findings were corrected for multiple comparison testing, the likelihood of making a type I error might not be completely disregarded and future studies should confirm our findings.

Several strengths of this study are worth considering. A detailed definition of the dietary habits and a valid assessment of the MD diet adherence was employed. Furthermore, psychological ill-being and well-being indicators were assessed during the second and the third trimester of pregnancy which provides a more comprehensive understanding of mental health along the pregnancy course.

## Conclusion

A greater MD adherence during gestation was associated with lower negative affect, depressive symptoms and anxiety, and with higher emotional regulation, resilience, and positive affect along the pregnancy course. A higher intake of wholegrain cereals, fruits, vegetables, fish, olive oil and nuts, together with a lower intake of red meat and sweets seemed to explain the observed associations of MD adherence with mental health indicators. Therefore, our findings suggest that the promotion of a higher diet quality during pregnancy might be effective to prevent mental health issues in pregnant women, yet this should be further tested by diet interventions in well-designed randomized controlled trials.

## Supplementary Information

Below is the link to the electronic supplementary material.

Supplementary file1 (DOCX 432 kb)
