# Supplementary material for: Associations of Mediterranean diet with psychological ill-being and well-being throughout the pregnancy course: The GESTAFIT project
Source: Qual Life Res. 2022 Mar 16;31(9):2705–16. doi: 10.1007/s11136-022-03121-2 (PMC9356938; doi:10.1007/s11136-022-03121-2)
Supplement: Supplementary file 1 — Supplementary file1 (DOCX 432 kb) [file 11136_2022_3121_MOESM1_ESM.docx]

**Supplementary Material**

Supplement to: “**Associations of Mediterranean diet with psychological ill-being and well-being throughout the pregnancy course. The GESTAFIT Project”.**

**Table of contents**

**Supplementary Figure S1**. Flow diagram of the study participants.

**Supplementary Table S1.** Inclusion and exclusion criteria in the GESTAFIT project.

**Supplementary Table S2.** Longitudinal associations of Mediterranean diet adherence at the 16^th^ gestational week with psychological ill-being and psychological well-being at the 34^th^ gestational week in the control group.

**Supplementary Table S3.** Differences in psychological ill-being and psychological well-being of pregnant women at the 16^th^ and 34^th^ gestational weeks by number of miscarriages (no previous miscarriages versus one or more miscarriages).


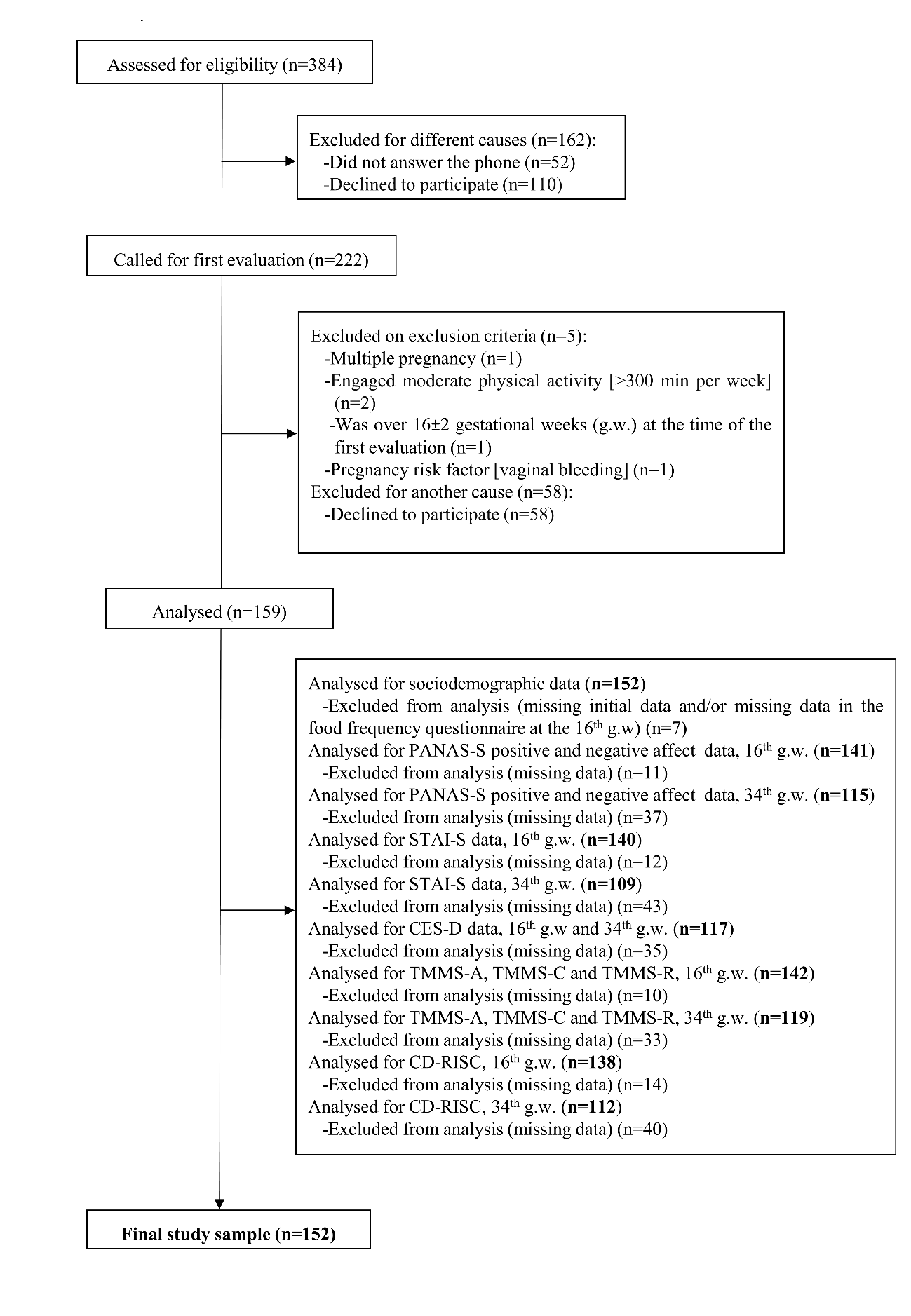


**Supplementary Figure S1**. Flow diagram of the study participants.

**Supplementary Table S1.** Inclusion and exclusion criteria in the GESTAFIT project.

| **Inclusion criteria** |
| --- |
| - Pregnant women aged 25-40 years old with a normal pregnancy course. |
| - Answering “no” to all questions on the PARmed-X for pregnancy.  - Being able to walk without assistance.  - Being able to read and write properly.  - Informed consent: Being capable and willing to provide written consent. |
| **Exclusion criteria** |
| - Having acute or terminal illness.  - Having malnutrition.  - Being unable to conduct tests for assessing physical fitness or exercise during pregnancy.  - Having pregnancy risk factors (such as hypertension, type 2 diabetes, etc.).  - Having a multiple pregnancy.  - Having chromosopathy or foetal malformations.  - Having uterine growth restriction.  - Having foetal death.  - Having upper or lower extremity fracture in the past 3 months.  - Suffering neuromuscular disease or presence of drugs affecting neuromuscular function.  - Being registered in another exercise program.  - Performing more than 300 minutes of at least moderate physical activity per week.  -Being engaged in another physical exercise program  - Being unwilling either to complete the study requirements or to be randomized into the control or intervention group. |

| **Supplementary Table S2.** Cross-sectional associations of Mediterranean diet adherence at the 16^th^ gestational week with psychological ill-being and psychological well-being at the 34^th^ gestational week in the control group. | | | | | | |
| --- | --- | --- | --- | --- | --- | --- |
|  | Longitudinal (16^th^ vs 34^th^ g.w.) | | | | | |
|  |  | Model I | |  | Model II | |
| **Mental health indicators** | n | β | p |  | β | p |
| *Psychological ill-being* |  |  |  |  |  |  |
| Negative Affect | 51 | -0.417 | **0.002** |  | -0.391 | **0.007** |
| Anxiety | 46 | -0.573 | **<0.001** |  | -0.583 | **<0.001** |
| Depression | 51 | -0.305 | **0.030** |  | -0.309 | **0.018** |
| *Psychological well-being* |  |  |  |  |  |  |
| Emotional Attention | 53 | -0.544 | 0.589 |  | 0.005 | 0.969 |
| Emotional Clarity | 53 | 0.195 | 0.162 |  | 0.210 | 0.150 |
| Emotional Regulation | 53 | 0.272 | **0.049** |  | 0.287 | **0.016** |
| Resilience | 48 | 0.352 | **0.014** |  | 0.334 | **0.034** |
| Positive Affect | 51 | 0.212 | 0.136 |  | 0.252 | 0.071 |
| Model I was unadjusted. Model II was adjusted for age, educational status, number of miscarriages, low back pain.  Boldface indicates statistical significance (*p*<0.05) | | | | | | |

| **Supplementary Table S3.** Differences in psychological ill-being and psychological well-being of pregnant women at the 16^th^ and 34^th^ gestational weeks by number of miscarriages (no previous miscarriages versus one or more miscarriages). | | | | | |  |
| --- | --- | --- | --- | --- | --- | --- |
| **Mental health indicators** | **n** | **No previous miscarriages** |  | **Previous miscarriages** | **p** | |
| *Psychological ill-being (16^th^ g.w.) ^a^* |  |  |  |  |  | |
| Negative Affect | 142 (82 vs 60) | 16.64 (0.75) |  | 18.40 (0.88) | 0.137 | |
| Anxiety | 141 (81 vs 60) | 13.54 (0.99) |  | 15.13 (1.16) | 0.306 | |
| Depression | 118 (71 vs 47) | 10.99 (0.98) |  | 11.99 (1.21) | 0.526 | |
| *Psychological ill-being (34^th^ g.w.) ^b^* |  |  |  |  |  | |
| Negative Affect | 115 (69 vs 46) | 17.99 (0.85) |  | 19.56 (1.05) | 0.253 | |
| Anxiety | 109 (65 vs 44) | 17.74 (1.38) |  | 15.87 (1.69) | 0.402 | |
| Depression | 117 (70 vs 47) | 13.86 (0.90) |  | 12.4 (1.10) | 0.311 | |
| *Psychological well-being (16^th^ g.w.) ^a^* |  |  |  |  |  | |
| Emotional Regulation | 143 (85 vs 58) | 29.98 (0.57) |  | 30.0 (0.69) | 0.984 | |
| Resilience | 138 (82 vs 56) | 30.41 (0.59) |  | 29.91 (0.71) | 0.587 | |
| Positive Affect | 142 (82 vs 60) | 34.23 (0.69) |  | 34.29 (0.81) | 0.955 | |
| *Psychological well-being (34^th^ g.w.) ^b^* |  |  |  |  |  | |
| Emotional Regulation | 119 (70 vs 49) | 29.54 (0.59) |  | 30.93 (0.71) | 0.136 | |
| Resilience | 112 (67 vs 45) | 30.22 (0.64) |  | 29.89 (0.78) | 0.752 | |
| Positive Affect | 115 (69 vs 46) | 32.67 (0.89) |  | 33.43 (1.09) | 0.598 | |
| Values shown as mean (standard error). ^a^Analyses were adjusted for age, educational status, number of miscarriages and low back pain. ^b^Analyses were additionally adjusted for exercise intervention.  Boldface indicates statistical significance (*p*<0.05) | | | | | |  |
